# Medication use and factors associated with opiate use among patients with diagnosed fibromyalgia from two ethnic sectors in southern Israel

**DOI:** 10.1186/s40545-023-00586-5

**Published:** 2023-06-26

**Authors:** Yulia Treister-Goltzman, Roni Peleg, Iftach Sagy, Idan Menashe

**Affiliations:** 1 Department of Family Medicine and Siaal Research Center for Family Practice and Primary Care, The Haim Doron Division of Community Health, Faculty of Health Sciences, Ben-Gurion University of the Negev, P.O. Box 653, 84105 Beer-Sheva, Israel; 2 Clalit Health Services, Southern District, Israel; 3 Rheumatology Disease Unit, Soroka University Medical Center, Beer-Sheva, Israel; 4 Clinical Research Center, Soroka University Medical Center, Beer-Sheva, Israel; 5 Faculty of Health Sciences, Ben-Gurion University of the Negev, Beer-Sheva, Israel; 6 Department of Public Health, Faculty of Health Sciences, Ben-Gurion University of the Negev, Beer-Sheva, Israel

**Keywords:** Fibromyalgia, Drug therapy, Opiate use, Analgesics, Ethnic differences

## Abstract

**Background:**

Our aims were to compare fibromyalgia (FM) rate, drug treatment and factors associated with the use of opiates in two ethnic sectors.

**Methods:**

A retrospective cross-sectional study in southern district of Israel was performed on diagnosed FM patients in 2019–2020 [7686 members (1.50%)]*.* Descriptive analyses were conducted and multivariable models for the use of opiates were developed.

**Results:**

There were significant differences between the two ethnic groups in FM prevalence at 1.63% and 0.91% in the Jewish and Arab groups, respectively. Only 32% of the patients used recommended medications and about 44% purchased opiates. Age, BMI, psychiatric co-morbidity, and treatment with a recommended drug were similarly associated with an increased risk for opiate use in both ethnic groups. However, male gender was associated with × 2 times reduced risk to use opiates only among the Bedouins (aOR = 0.552, 95%CI = 0.333–0.911). In addition, while in both of ethnic groups the existence of another localized pain syndrome was associated with an increased risk for opiates use, this risk was 4 times higher in the Bedouin group (aOR = 8.500, 95%CI = 2.023–59.293 and aOR = 2.079, 95%CI = 1.556–2.814).

**Conclusions:**

The study showed underdiagnosis of FM in the minority Arab ethnicity. Female Arab FM patients in low or high, compared to middle socio-economic status, were a risk group for excess opiate use. Increased use of opiates and very low rate of purchase of recommended drugs point to a lack of effectiveness of these drugs. Future research should assess whether the treatment of treatable factors can reduce the dangerous use of opiates.

## Background

Because of the complexity of fibromyalgia (FM) and limited understanding of its mechanism, there is no effective treatment and a broad range of pharmacological and non-pharmacological treatments are used [1]. Several drugs are approved today for the treatment of FM and are recommended by the Israel Rheumatology Association [2]. Despite evidence against the use of strong opiates for the treatment of FM, their use has increased over recent years leading to potential addiction and even death from overdose [3].

A very small number of studies have focused on ethnic differences in FM. Although FM is most often diagnosed in middle-age White women [4], many researchers contend that the lower prevalence among minority ethnic groups is due to a low diagnosis rate and, possibly, a lower degree of understanding of and belief in the patient’s complaints by the medical team [4]. Members of minority groups tend to have lower educational and socio-economic levels and demonstrate lower help-seeking behavior, which also contributes to underdiagnosis [5]. In recent years, there have been reports of a higher prevalence of FM in ethnic minorities [6–8]. The clinical manifestations of FM and relevant coping strategies are different in ethnic minorities [9]. A study in Israel compared the severity of FM among women of Sephardic and Ashkenazi origin and found that symptoms were more severe in the Sephardic group, but ascribed this finding to the lower educational level among Sephardic women [10].

Two main ethnic groups live in southern Israel, with Jews comprising 75% and Bedouin-Arabs 25%. On the whole, the Jewish population follows a western lifestyle. The Bedouin population is an Arab Muslim population with authentic customs and traditions. It is ranked at the lowest socio-economic status in Israel and characterized by a low educational level [11]. As there are differences in genetic background, lifestyle, and socio-economic status between these populations, it is reasonable to expect differences in various characteristics of FM patients and in the drugs purchase. The aim of the present study was to compare medication use among Bedouin and Jewish FM patients in southern Israel and to identify factors associated with opiates use.

## Methods

### Study design

We conducted a retrospective cross-sectional study in a population of all FM patients 18–90 years of age, who are members of the Clalit Healthcare Services (CHS), southern district.

Since 1994 a compulsory national health insurance law has provided the entire population of Israeli citizens with a diverse basket of healthcare services and drugs. The majority Jewish and the minority Arab populations, are covered by exactly the same medication options and health services, provided in local outpatient clinics and regional hospitals [12]. Opiate analgesics are relatively available in Israel: as a part of the government-subsidized Israeli Health Basket, any physician and pharmacy can prescribe and dispense them [13].

The CHS is the largest health maintenance organization (HMO), covering over 52% of the population and > 70% of the residents in southern Israel. The CHS has a validated, centralized computerized databased system that contains clinical, economic, and administrative data for its entire roster of insured individuals. For this study, we used data that were obtained from the CSH electronic database between January 1, 2019 and December 31, 2020. FM patients were defined as patients whose FM diagnosis was confirmed by a rheumatologist (ICD-9-7291) prior to the study’s starting date (January 1, 2019). Patients whose diagnosis was not confirmed by a rheumatologist were excluded from the study.

### Data collection

The following variables on the FM patients were obtained from the CHS database:

- *Socio-demographic data*—age, sex, ethnicity, country of birth, year of immigration to Israel, socio-economic status (based on site of residence as listed in the database).
- *Relevant chronic co-morbidity—all morbidity relevant to FM was recorded*
  - Physical—localized pain syndromes including low back pain and temporomandibular pain, and systemic rheumatic diseases including rheumatoid arthritis, spondylarthropathies, and diffuse connective tissue diseases. Psychiatric conditions including depression, anxiety, and post-traumatic stress disorder
  - The Charlson Co-morbidity Score, which is used to predict annual mortality based on adjusted significant chronic co-morbidity [14]. The total score ranges from 10 to 37.
- *The use of relevant drugs* was determined by actual purchase in the pharmacy. The purchase of at least two physicians’ prescriptions for a drug was considered evidence of use. The drugs used for FM were divided into two groups:
  - Drugs recommended by the Israeli Rheumatology Society and approved for use (recommended drugs): amitriptyline, duloxetine, pregabalin, gabapentin, and milnacipran.
  - Widely used analgesic drugs including paracetamol, tramadol, non-steroidal anti-inflammatory drugs (NSAIDs), and opiates.
- *The duration of disease, in years*, from the time the diagnosis of FM was first listed the medical record and up to January 1, 2019, the date of entry into the study.
- *Other data from the medical record*: BMI.
- *The total number of CHS members* aged 18–90 years in the two ethnic sectors in the study period.

### Statistical analyses

Statistical analyses were conducted with the R software (version 4.0.5). Qualitative variables are presented as frequency and percentages. Continuous variables are presented as mean with standard deviation, or median with range, as appropriate for the distributions. Differences in continuous variables were assessed with the t-test or the Mann–Whitney test. Differences in nominal variables were tested with the Chi-square test or the Fisher exact test. Logistic regression models were built for the use of opiates for each ethnic sector separately. All independent variables were tested for association with the dependent variable in univariate analyses. Because of potential interactions and confounding, any independent variable found to have an association with a statistical significance of *p* ≤ 0.2 for opiate use was entered into the multivariable regression analysis. The independent variables were tested for collinearity using appropriate tests. Interactions and confounding were tested for each couple of independent variables with a statistically significant association between them. Finally, Breslow–Day test for homogeneity of odds ratios was performed for dichotomous variables in two ethnic groups.

The Ethics Committee of CHS approved the study, and exempted it from the requirement to obtain informed consents (#COM2-0213-20).

## Results

### Background characteristics of the patients

The patients’ background characteristics are presented in Table 1. During the study period there were 513,889 insured individuals in the southern district of CHS between the ages of 18–90. Of these, 7686 (1.5%) were diagnosed with FM; 0.91% in the Arab and 1.63% in the Jewish ethnic groups (*p* < 0.0001). There were statistically significant differences between the ethnic groups in most background characteristics. The Arabs were younger with a mean age of 49.20 years compared to 55.42 years in the Jewish group. A lower percentage of Arabs were born abroad (9.52% vs. 44.29% among Jews, *p* < 0.0001). The socio-economic status of the Arabs was lower with 67.43% in the low class compared to 32.87% among Jews (*p* < 0.0001). The Arabs had a higher BMI than the Jews at 30.11 vs. 28.04, respectively (*p* < 0.0001). A high percentage of Arab participants had another localized pain disorder and/or a psychiatric disorder at 97.94% vs. 94.69% and 57.34% vs. 61.70%, respectively (both with *p* < 0.0001). Conversely, Jewish participants had a higher Charlson score with a median of 2 compared to 1 among the Arabs (*p* < 0.0001).

**Table 1:** Baseline characteristics of the patient population

Variable	Total	Arabs	Jews	*p*-value
Fibromyalgia diagnosis *N* (%)	7686 (1.50)	872 (0.91)	6814 (1.63)	**< 0.0001**^a^
Age				
Mean (SD)	54.71 (13.80)	49.20 (10.97)	55.42 (13.96)	**< 0.0001**^b^
Median (IQR^¶^)	56.00 (45–64)	49.00 (42–56)	57.00 (45–65)	
Gender (female) *N* (%)	6901 (89.79)	769 (88.19)	6131 (89.99)	0.111^a^
Immigrant *N* (%)	3101 (40.35)	83 (9.52)	3018 (44.29)	**< 0.0001**^a^
Years since immigration to Israel				
< 5 years	17 (0.55)	0 (0)	17 (0.56)	0.765^a^
5–10 years	40 (1.29)	0 (0)	40 (1.33)	
> 10 years	3044 (98.16)	83 (100)	2961 (98.11)	
Socio-economic level *N* (%)				
Low	2828 (36.79)	588 (67.43)	2240 (32.87)	**< 0.0001**^a^
Middle	2486 (32.34)	34 (3.90)	2452 (35.98)	
High	2372 (30.86)	250 (28.67)	2122 (31.14)	
Disease duration (years) Median (IQR^¶^)	7.84 (3.23–12.03)	8.06 (3.07–13.20)	7.83 (3.25–11.91)	0.097^c^
BMI				
Median (IQR^¶^)	28.23 (24.56–32.59)	30.11 (26.49–34.36)	28.04 (24.34–32.39)	**< 0.0001**^c^
Missing values *N* (%)	562 (7.31)	83 (9.52)	479 (7.03)	**0.010**^a^
Other pain syndrome *N* (%)				
At least one other localized pain syndrome	7306 (95.06)	854 (97.94)	6452 (94.69)	**< 0.0001**^**a**^
Low back pain	7229 (94.05)	849 (97.36)	6380 (93.63)	**< 0.0001**^**a**^
Temporomandibular pain	1892 (24.62)	251 (28.78)	1641(24.08)	**0.003**^**a**^
Autoimmune inflammatory rheumatic disease *N* (%)	1244 (16.19)	125 (14.33)	1119 (16.42)	0.127^a^
Psychiatric co-morbidity *N* (%)				
At least one other psychiatric disorder	4704 (61.20)	500 (57.34)	4204 (61.70)	**0.014**^**a**^
Anxiety and/or depression	4594 (59.77)	497 (57.00)	4097 (60.13)	0.082^a^
Posttraumatic stress disorder	586 (7.62)	27 (3.10)	559 (8.20)	**< 0.0001**^**a**^
Charlson co-morbidity score Median (IQR^¶^)	2.00 (1–4)	1 (0–3)	2 (1–4)	**< 0.0001**^**c**^

^a^Chi-square/Fisher test, ^b^t-test, ^c^Mann–Whitney test

^¶^Interquartile range

Bold values indicate significance

### Use of drugs

**Fig. 1:**
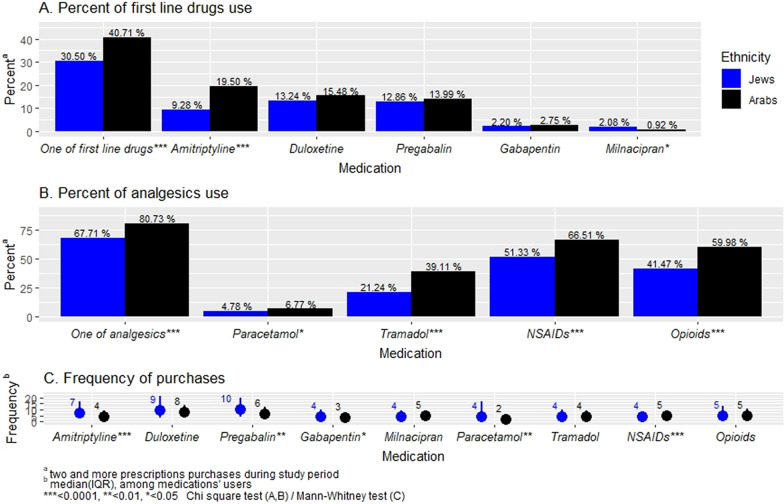
Medication use among fibromyalgia patients in southern Israel

Figure 1 shows the rate of drugs use by FM patients in the study population. As mentioned above, the use of drugs was defined as the purchase of at least two prescriptions for a drug over the study period. About one-third of the FM patients used at least one of the first-line drugs recommended by the Israel Rheumatology Association with a higher rate in the Arab group (40.75% vs. 30.5%, *p* < 0.0001) (Fig. 1A). The most commonly used drug was duloxetine (13.49%), followed by pregabalin (12.98%), and amitriptyline (10.43%). Negligible use rates were found for gabapentin and milnacipran. The use of amitriptyline was significantly higher in the Arab sector (19.50% vs. 9.28%, *p* < 0.0001) and of milnacipran in the Jewish sector (2.08% vs. 0.92%, *p* < 0.05). In addition, over 69% of the patients used at least one analgesic drug, with over 53% used NSAIDs, about 44% used an opiate, over 23% used the opiate analog tramadol, and only 5% used paracetamol. The use of all these analgesic drugs was significantly higher in the Arab group (Fig. 1B).

Figure 1C shows the median and interquartile range of drug purchases among FM patients who used the drugs from two ethnic groups. The highest level of adherence to drugs among users was for pregabalin and duloxetine (median = 9). The median for dispensing of amitriptyline was 6 and for milnacipran and gabapentin 4. The adherence to pregabalin, amitriptyline, and gabapentin was significantly higher in the Jewish sector (*p* < 0.05). The highest adherence for analgesics was for opiates with a median of five purchases without a significant difference between the ethnic groups. Nevertheless, Arab patients used to purchase NSAIDs more frequently than Jews (median = 5 vs. 4, respectively, *p* < 0.0001).

### Factors associated with the use of opiates

In the univariate tests use of opiates was significantly associated with the ethnicity, age, socio-economic status, the duration of disease, BMI, another localized pain syndrome, a rheumatic disease, a psychiatric disease, the Charlson score, and treatment with recommended drugs of the patients (for all *p* < 0.0001).

Examining the independent association of these factors in multivariate logistic regression models in the two ethnic sectors (Table 2) revealed that age (aOR = 1.013, 95% CI = 1.007–1.019 and aOR = 1.063, 95% CI = 1.041–1.087), BMI (aOR = 1.021, 95% CI = 1.012–1.030 and aOR = 1.051, 95% CI = 1.022–1.082), psychiatric co-morbidity (aOR = 1.168, 95% CI = 1.045–1.306 and aOR = 1.334, 95% CI = 0.962–1.852) and treatment with a recommended drug (aOR = 2.835, 95% CI = 2.530–3.179 and aOR = 3.104, 95% CI = 2.220–4.376) were similarly associated with an increased risk for opiate use in both the Jewish and Bedouin sectors, respectively. However, male gender was associated with × 2 times reduced risk to use opiates only among the Bedouins (aOR = 0.552, 95% CI = 0.333–0.911). In addition, while in both ethnic groups the existence of another localized pain syndrome was associated with an increased risk for opiates use, this risk was 4 times higher in the Bedouin group (aOR = 8.500, 95% CI = 2.023–59.293 and aOR = 2.079, 95% CI = 1.556–2.814).

**Table 2:** Models for opiate use among fibromyalgia patients from two ethnic groups (*N* = 7686)

Variable	Jewish ethnicity		Arab ethnicity	
	OR	CI (95%)	OR	CI (95%)
Age	1.013***	1.007–1.019	1.063***	1.041–1.087
Gender (male)	1.004	0.836–1.206	0.552*	0.333–0.911
Socio-economic level				
High (reference)	–	–	–	–
Middle	1.128	0.990–1.286	0.338*	0.142–0.788
Low	1.368***	1.197–1.563	1.141	0.791–1.642
Disease duration	0.991	0.980–1.002	1.002	0.971–1.035
BMI	1.021***	1.012–1.030	1.051***	1.022–1.082
Another localized pain syndrome	2.079***	1.556–2.814	8.500**	2.023–59.293
Psychiatric co-morbidity	1.168**	1.045–1.306	1.334	0.962–1.852
Charlson score	1.104***	1.069–1.141	0.934	0.830–1.053
Treatment with a recommended drug	2.835***	2.530–3.179	3.104***	2.220–4.376

***p < 0.001, **p < 0.01, *p < 0.05

Table 3 shows the results of the Breslow–Day test for homogeneity of odds ratios in two ethnic groups. Heterogenous odds ratios for gender and another localized pain syndrome indicate interaction of these variables with the ethnic group.

**Table 3:** Associations of dichotomic independent variables with opiates use in two ethnic groups

Variable	Jewish ethnicity		Arab ethnicity		Homogeneity of OR^a^, *p*-value^c^
	OR^a^	CI (95%)^b^	OR^a^	CI (95%)^b^	
Gender (male)	0.994	0.846–1.167	0.494***	0.325–0.747	0.002
Another localized pain syndrome	3.054***	2.360–4.005	12.517***	3.532–79.497	0.048
Rheumatic disease	1.503***	1.322–1.709	1.864**	1.240–2.860	0.332
Psychiatric co-morbidity	1.442***	1.304–1.594	1.569**	1.193–2.065	0.569
Treatment with a recommended drug	3.241***	2.913–3.609	3.393***	2.523–4.594	0.778

^a^Odds ratio, ^b^confidence interval (95%), ^c^Breslow–Day test, ***p < 0.001, **p < 0.01

## Discussion

### Characteristics of patients diagnosed with FM in southern Israel

The prevalence of FM in patients aged 18–90 years found in this study was 1.5%, a slightly higher prevalence than reported in another study from the US (1.1%) that used the same diagnostic criteria (i.e., FM recorded in the medical record and confirmed by a consulting rheumatologist) [15]. Higher prevalence rates of FM were reported in self-report/population surveys in Israel 2.0%-2.6% [16] as well as elsewhere [15, 17, 18]. Yet, it is expected that the estimates of FM prevalence in self-reported surveys will be higher than its actual diagnosis rate in the population. The significantly lower prevalence of FM in the Bedouin group compared to the Jewish is not surprising given the reported low prevalence rates of FM in minority groups in other countries [4, 19]. A possible underdiagnosis of FM in the Bedouins could be due to misinterpretation of the medical staff of the clinical symptoms associated with FM in this sector [4, 8, 10]. Indeed, a study from southern Israel showed that the rate of agreement between family doctors and rheumatologists on the diagnosis of FM was better in the Jewish ethnic group (Kappa 0.7) than in the Arab (Kappa 0.3) [20]. The differences in socio-economic characteristics between FM patients in the two sectors are not unique to FM patients and represent the general socio-economic differences between these two ethnic groups in southern Israel [5, 11, 21, 22].

The overlap between FM and other localized pain syndromes, rheumatic diseases and psychiatric co-morbidity is known and covered in the medical literature [6, 17, 23–31].

The higher Charlson score in the Jewish sector can be explained by the older age of this population and the higher incidence of malignancy in this group [32].

### Medications use and factors associated with opiates use

The rate of recommended drugs purchase in this study was relatively low with approximately third of the patients purchased any kind of drug over the study period. However, this rate was similar to the reported in other studies from the United States [33–35] and from Israel [34]. The highest purchase rate for recommended drugs in our study was for duloxetine, a drug that is known for its relatively high adherence rate [33, 35–38]. The finding of negligible use of milnacipran also has been reported in other studies [33, 35].

Our study showed that the use of the recommended drugs and analgesics was significantly higher in the Arab group. Very few studies have evaluated the association between ethnic origin and adherence to treatment in FM. A study from the United States showed that Black patients are more adherent than Whites [33]. While no Israeli study has investigated a possible association between ethnic origin and purchase of drugs for FM, studies on other conditions showed that Arabs purchased drugs more than Jews [21, 39].

It appears that due to a low level of satisfaction with treatment with recommended drugs there is significant use of analgesic drugs, including less recommended drugs such as NSAIDs (above 53%), that have the potential to cause cardiovascular and gastrointestinal injury with prolonged use [40], and opiates (above 43%), that are addictive and have become an epidemic in some countries [41, 42]. A study from Spain, based on a telephone interview, found a usage rate of 16% for NSAIDs and 7.5% for opiates [43]. Studies based on actual purchases reported usage rates of 56–68% for NSAIDs and 14–56% for opiates [44–48]. Our findings show a higher rate of opiate use among FM patients in southern Israel than in the rest of the country [48] and one of the highest rates reported in the literature. In the present study, we tested many potential predictor variables to identify possible associations with the opiates use among FM patients. Some of the factors that were associated with opiate use (age, socio-economic status, Charlson score) are not treatable, but their recognition could increase our understanding and provide explanations for the increased use of opiates. In contrast, physicians can affect and possibly reduce the exaggerated use of opiates by relating to treatable factors including high BMI, an additional localized pain syndrome, and psychiatric co-morbidity. Only one study was found in the literature that tried to identify factors associated with treatment with opiates among FM patients. Duration of disease, age, and severity of pain were estimated risk factors [45], providing support for the findings in the present study.

The combination of low use of recommended drugs and the association between this with opiates use may indicate a low level of effectiveness of the existing drug options for FM and an urgent need to develop new and more effective drugs. These assumptions are supported by the findings of the additional studies, which show a higher level of satisfaction among patients with non-pharmacological treatment [49], and studies that provide objective proof of physical and emotional improvement among FM patients with multidisciplinary treatment [50], psychology-based treatment and relaxation [51, 52]. To our knowledge, this is the first study that evaluated ethnic differences in the use of opiates among FM patients. Male sex and a middle vs. high socio-economic status were protective estimators of opiate use in the Arab sector only. Thus, the group for which special care should be taken in prescribing opiates is female Arab FM patients of low and high socio-economic status.

The strength of the association between an additional localized pain syndrome and the use of opiates in the Arab group (OR = 8.50) is particularly striking. Addressing this co-morbidity could reduce the use of opiates in the Arab sector. In the Jewish group, patients of low socio-economic status who use one of the recommended drugs for FM are at risk for excessive use of opiates.

### Study limitations

Because of low availability of rheumatologists in southern Israel some patients may go for private consultations with rheumatologists and would not, therefore, be recorded in the CHS database or included in the study population. In that case, there could be an underrepresentation of patients in the higher socio-economic status, who may behave differently than the others in terms of drug treatment and the use of opiates.

The sample of Arab patients who were diagnosed with FM was relatively small. It is possible that only those with high help-seeking behavior or more severe symptoms were diagnosed, which could selectively increase the measures of association in this population. On the other hand, an overall lower socio-economic status in this population could impact the ability to purchase first-line or analgesic (including opiates) medications, despite a subsidized price.

There is also a generic issue of missing data in computerized medical records, which is typical of studies based on these records. For example, BMI was missing in 7% of the records, an issue that could affect the final results of the models. Similarly, since the study was based on data only, we could not assess patients’ personality characteristics that also might be associated with the use of opiates.

## Conclusions

The present study, the first to characterize drug treatment and identify unique factors associated with the use of opiates in FM patients from two ethnic origins in southern Israel, identified unique factors associated with the use of opiates in each sector. The results indicate underdiagnosis of FM in the minority Arab ethnicity and a high rate of opiate use among FM patients, which should serve as a warning for healthcare providers. The results also showed that Arab women in the lower and higher vs. the middle socio-economic levels are at risk for excessive opiate use. The combination of increased use of opiates with the very low rate of purchase of recommended drugs may point to a lack of effectiveness of these drugs and indicates the urgent need to develop and implement more effective therapeutic approaches. The finding that the presence of co-morbid localized pain syndromes and psychiatric diseases increases the opiate use among FM patients is potentially treatable. The findings of this study can help healthcare providers direct their efforts to the treatment of the unique factors in each ethnic sector. Future studies can prove whether addressing these risk factors actually reduces opiate use.

## Data Availability

The data that support the findings of this study are available from “Clalit Health Services”, but restrictions apply to the availability of these data, which were used under license for the current study, and so are not publicly available. Data are however available from the authors upon reasonable request and with permission of “Clalit Health Services”.

## Abbreviations

FM: Fibromyalgia
CHS: Clalit Healthcare Services
NSAIDs: Non-steroidal anti-inflammatory drugs
BMI: Body mass index
